# Disposable Polydimethylsiloxane (PDMS)-Coated Fused Silica Optical Fibers for Sampling Pheromones of Moths

**DOI:** 10.1371/journal.pone.0161138

**Published:** 2016-08-17

**Authors:** Rik Lievers, Astrid T. Groot

**Affiliations:** 1 Institute for Biodiversity and Ecosystem Dynamics, University of Amsterdam, Science Park 904, Amsterdam, the Netherlands; 2 Max Planck Institute for Chemical Ecology, Hans-Knöll-Strasse 8, Jena, Germany; Fundacao Oswaldo Cruz, BRAZIL

## Abstract

In the past decades, the sex pheromone composition in female moths has been analyzed by different methods, ranging from volatile collections to gland extractions, which all have some disadvantage: volatile collections can generally only be conducted on (small) groups of females to detect the minor pheromone compounds, whereas gland extractions are destructive. Direct-contact SPME overcomes some of these disadvantages, but is expensive, the SPME fiber coating can be damaged due to repeated usage, and samples need to be analyzed relatively quickly after sampling. In this study, we assessed the suitability of cheap and disposable fused silica optical fibers coated with 100 μm polydimethylsiloxane (PDMS) by sampling the pheromone of two noctuid moths, *Heliothis virescens* and *Heliothis subflexa*. By rubbing the disposable PDMS fibers over the pheromone glands of females that had called for at least 15 minutes and subsequently extracting the PDMS fibers in hexane, we collected all known pheromone compounds, and we found a strong positive correlation for most pheromone compounds between the disposable PDMS fiber rubs and the corresponding gland extracts of the same females. When comparing this method to volatile collections and the corresponding gland extracts, we generally found comparable percentages between the three techniques, with some differences that likely stem from the chemical properties of the individual pheromone compounds. Hexane extraction of cheap, disposable, PDMS coated fused silica optical fibers allows for sampling large quantities of individual females in a short time, eliminates the need for immediate sample analysis, and enables to use the same sample for multiple chemical analyses.

## Introduction

To attract a potential mating partner, female moths emit a species-specific sex pheromone. Moth sex pheromones usually consist of long-chain fatty acid derivatives of various compound classes (such as alcohols, aldehydes, and acetate esters), which are produced in a specified gland at the tip of the female abdomen and released in specific ratios [1]. To determine the sex pheromone composition, glands are often destructively extracted by cutting off the gland and soaking it in organic solvent [2]. The ratios of the compounds found through this procedure may differ from the blend that is actually released by the female moth and thus to what the male is actually responding to [3].

Assessment of the emitted and behaviorally active pheromone compounds is often a cumbersome procedure, as moth pheromones are released in very small quantities [4,5]. Therefore, many studies on emitted volatiles have been conducted on pools of moths. Common methods to collect pheromones of pools of calling moths include the use of an airflow to trap the pheromone on porous polymer sorbents such as TENAX [6], Super-Q [7], or Porapak-Q [8]. Cardé et al. [9] collected volatiles by rinsing pheromone adsorbed to the inner walls of the glass flask that had contained the calling moths. The principle of using a glass surface to collect moth pheromone has been applied to collect pheromones from individual calling females as well [4,10,11]. Activated charcoal [5] and Porapak-Q [12] have also been used to collect pheromone from individual moths. A more recent innovative method uses a GC column [7].

Since the 1990s, Solid Phase Micro Extraction (SPME) has become increasingly popular for the sampling of volatile organic compounds. SPME was originally developed by Arthur and Pawliszyn [13] and uses a silica-fused fiber coated with a thin film of either a liquid polymer [e.g. polydimethylsiloxane (PDMS)] or a porous solid sorbent (e.g. carboxen; see review by Pawliszyn et al. [14]). Liquid sorbents extract analytes through absorption, whereas solid sorbents extract analytes through adsorption (see review by Pawliszyn et al. [14]). SPME has been used for sampling organic compounds from live organisms, including many insects [15,16]. For example, Borg-Karlson and Mozûraitis [17] sampled the pheromone released by individual female moths from headspace by keeping the SPME fiber a few millimeters from the gland of a calling female for 2–3 hours. For a more recent example of using SPME to collect moth pheromone from headspace see [18]. However, these methods are laborious, as the very low amount of pheromone released by moths requires long extraction times of several hours. Zhu et al. [19] collected pheromone directly from the gland surface by using a filter paper soaked in hexane. Frérot et al. [20] successfully collected pheromones by rubbing the surface of the gland with SPME fibers, leaving the animal intact. These so-called “direct-contact SPME” samples were qualitatively and quantitatively similar to solvent extractions. This method has since been used successfully in a number of studies using various types of coating: 100 μm PDMS- [21–24], 7 μm PDMS- [20,25], 65 μm CW/DVB- [26–33], and 50/30 μm DVB/CAR/PDMS-coated fibers [34]. Insect cuticular hydrocarbons have been sampled successfully this way as well [16].

However, there are some problems with the commercially available SPME fibers (Supelco, Bellefonte, PA, USA): a) they are expensive, b) repeated use could cause damage to the coating, and c) they have to be analyzed relatively quickly compared to other methods [35,36]. In addition, d) long GC analysis times limit the use of SPME fibers for collecting and analyzing large numbers of samples in a short amount of time, unless the fibers are extracted in organic solvent and reused [35]. Therefore, direct-contact SPME is not widely applied and has only been used for large sample sizes (N > 50) in a small number of studies [26,28,29,31,33]. To overcome the disadvantages, several groups have worked on alternatives to SPME to sample cuticular hydrocarbons, which include the use of silicone rubber tubing [36] and self-made uncoated glass fibers [37]. Mayer et al. [38] first exemplified the use of disposable fused silica optical fibers (for measuring water contaminants), which can be bought in bulk rolls that are normally used for data transmission, and can be cut into custom lengths. Currently, disposable fused silica optical fibers with two types of coating are commercially available at various coating thicknesses: Polydimethylsiloxane (PDMS) and Polyacrylate (PA; Polymicro Technologies Inc., Phoenix, AZ). Disposable usage of these fused silica optical fibers for sampling pheromone glands costs a fraction of the original material costs of SPME. Furthermore, analytes can be desorbed from the fiber using an organic solvent and stored until GC-analysis [35], without the need to worry about damaging the coating. This has the additional advantage that the same sample can be used for multiple analyses such as GC-EAD, GC-MS, or derivatization.

In this study, we assessed the suitability of disposable 100 μm PDMS-coated fused silica optical fibers (hereafter called disposable PDMS fibers) by sampling the female pheromone of two noctuid moths, *Heliothis virescens* (Hv) and *Heliothis subflexa* (Hs) (Lepidoptera, Noctuidae). The pheromone composition of these two species has been well characterized in the past decades (Hv: [4,5,39–42] and Hs: [5,41,43,44]). The female pheromone blend of these species contains saturated and unsaturated fatty acid derivatives of various lengths and of various compound classes (aldehydes, acetate esters, and alcohols), which represent a wide variety of compounds present in many other moth species as well. We sampled females of two different lines each of Hv and Hs that share most of the compounds in their blend, but vary extensively in both the absolute amounts and percentages of the compounds. This large variation allowed us to determine correlations of a large range of percentages of each compound between pheromone collections through a) disposable PDMS fibers, b) traditional solvent gland extracts, and c) volatile collections. We found that the disposable PDMS fibers are suitable for collecting secreted pheromone from the exterior of individual moth pheromone glands, and that the composition of these pheromone collections is similar to traditional solvent gland extracts and volatile collections.

## Materials and Methods

### Insects

To test the suitability of disposable PDMS fibers, we sampled female moths of four different laboratory populations of Hv and Hs (Table 1): we used two artificial selection lines of Hv that were constructed to have opposing ratios between the unsaturated and saturated sex pheromone compounds [45]. The selection line with high 16:Ald/Z11-16:Ald and 14:Ald/Z9-14:Ald ratios is referred to as Hv “High” (HvH). Females with low 16:Ald/Z11-16:Ald and 14:Ald/Z9-14:Ald ratios are referred to as Hv “Low” (HvL). For Hs, we also used two different lines. One line originated from the Hs rearing at North Carolina State University [46] to which newly collected larvae from organic tomatillo plantations in North Carolina were added in July 2014, in the following indicated as Hs. A second Hs line (HsDD23) was constructed by introgressing the quantitative trait locus (QTL) for non-acetate production of Hv, located on chromosome 22, into the genomic background of Hs [47]. The acetates of the resulting HsDD23 line comprised less than 3% of the total blend [48], compared to up to 42% that can be found in Hs glands [5,46,49]. In addition, HsDD23 contains significantly more Z11-16:OH than Hs without this introgressed QTL [47]. In all experiments, we used 2- to 8-day-old adult virgin female moths from these four different lines. The cultures were kept at 25°C and 60% relative humidity with a reversed 14h:10h day-night cycle (lights off at 11.00, on at 21.00). Hv larvae were maintained on a pinto bean based diet, and Hs larvae on a wheat germ/soy flour based diet (BioServ Inc., Newark, DE, USA). Adult moths were provided with a cotton roll soaked in 10% sugar water.

**Table 1:** Female pheromone composition of HvL, HvH, Hs and HsDD23.

Compound	Abbreviation	HvL (n = 28)	HvH (n = 30)	Hs (n = 49)	HsDD23 (n = 86)
tetradecanal	14:Ald	3.6 ± 0.4	18 ± 1	0.2 ± 0	0.4 ± 0
(Z)-9-tetradecenal	Z9-14:Ald	6.3 ± 0.4	0.1 ± 0	0.3 ± 0	0.7 ± 0
hexadecanal	16:Ald	6.7 ± 0.5	80 ± 1	2.4 ± 0.1	6.1 ± 0.3
(Z)-7-hexadecenal	Z7-16:Ald	2.1 ± 0.3	0.7 ± 0.1	0.5 ± 0	0.6 ± 0
(Z)-9-hexadecenal	Z9-16:Ald	0.6 ± 0.1	0.5 ± 0	21 ± 0	14 ± 0
(Z)-11-hexadecenal	Z11-16:Ald	69 ± 1	0.5 ± 0.1	49 ± 1	54 ± 1
(Z)-7-hexadecenyl acetate	Z7-16:OAc	-	-	0.5 ± 0.1	0.1 ± 0
(Z)-9-hexadecenyl acetate	Z9-16:OAc	-	-	4.0 ± 0.2	0.2 ± 0
(Z)-11-hexadecenyl acetate	Z11-16:OAc	-	-	11 ± 1	1 ± 0.1
(Z)-9-hexadecen-1-ol	Z9-16:OH	-	-	1.5 ± 0.1	-
(Z)-11-hexadecen-1-ol	Z11-16:OH	12 ± 1	0.2 ± 0	9.4 ± 0.3	23 ± 1

Percentage (mean ± SEM) of each pheromone compound in gland extracts of HvL (data from [45]), HvH (data from [45]), Hs (data from [46]) and HsDD23 (data from [48]).

- indicates that the pheromone compound was absent or not (reliably) detected.

### Disposable PDMS fibers

To determine the composition of the pheromone blend collected from the gland surface, a fused silica optical fiber coated with a 100 μm PDMS layer (Polymicro Technologies Inc., Phoenix, AZ, USA) was cut into 15 mm pieces using a scalpel. The fibers were handled with smooth tip forceps (KFI y. k. tweezers, K-7 No.J 18–8 Stainless Steel; KFI, Japan). Shorter pieces (e.g. 10mm) would be sufficient for collecting pheromone, however, 15 mm pieces allowed a better grip with the forceps. After cutting, the fibers were placed in a stainless steel rack in a specifically designed glass conditioning unit which was originally developed for using disposable PDMS fibers to sample polycyclic aromatic hydrocarbons from sedimentary pore waters [50]. The rack holding the fibers was designed such that the fibers did not come into direct contact with any surface. The glass conditioning unit was placed into an N_2_ flow in the oven of an adapted HP5890A gas chromatograph (Hewlett-Packard, Palo Alto, CA, USA) (S1 Fig). The conditioning protocol was as follows: 35°C (hold for 1 minute), followed by an increase to 200°C (20°C/minute, hold for 40 minutes). After conditioning, the fibers were stored in an air-tight glass container (S2 Fig) at room temperature until use.

PDMS is known to absorb pheromone compounds with different degrees of efficiency [51], i.e. some compounds can be absorbed by the fiber coating faster than others. Therefore, we first tested the reproducibility of the method by absorbing known amounts of a mixture of synthetic pheromone compounds (obtained from Pherobank, Wijk bij Duurstede, The Netherlands) from aluminum foil. We pipetted 2 μL of hexane (Rotisolv HPLC; Carl Roth, Karlsruhe, Germany) containing a total of about 330 ng of the synthetic pheromone blend onto the foil. The blend consisted of approximately equal ratios of pheromone compounds. After the hexane was evaporated, the foil was rubbed with the fiber for 1, 2, or 4 minutes. Each fiber was subsequently placed in a glass micro-volume vial insert (self-made from 150 mm VOLAC glass pasteur pipettes) containing 50 μL hexane and 200 ng pentadecane as internal standard. As a control, 2 μL of the synthetic pheromone blend was added directly to 50 μL hexane with internal standard. The inserts were placed in a 4 mL screw neck vial (Grace Discovery Sciences, Columbia, MD, USA) and capped with a solid top polypropylene cap with a TFE (tetrafluoroethylene)⁄silicone-bonded interseal (Grace Discovery Sciences). The fibers were then rinsed by gently tilting the insert a couple of times. After 30–60 minutes, fibers were rinsed again and removed from the insert and extracts were stored at -20°C until analysis (not longer than 2 weeks).

Secreted pheromone present on the gland surface was collected 4–8 hours into scotophase. After observing females calling for at least 15 minutes, glands were extruded by gently squeezing the abdomen with forceps (Dumont #55 forceps INOX; Fine Science Tools, Heidelberg, Germany) equipped with two cotton rolls (S3 Fig). Glands were rubbed with the fiber for at least 2 minutes, using all sides of the fiber. The pheromone was then desorbed from the fiber in hexane, as described above. We refer to these collections of secreted pheromone as “PDMS rubs”. To confirm that the fibers were clean, we extracted blank fibers as control.

### Gland extracts

To determine the relationship between PDMS rubs and the gland extracts, the rubbed glands were excised directly after the PDMS rubs. Female glands were extruded by gently squeezing the abdomen with three fingers. The extruded glands were fixed by firmly holding the abdomen with forceps (Dumont #55 forceps INOX; Fine Science Tools) just anterior of the gland and excised with microdissection scissors (Vannas-Tübingen spring scissors, 5 mm Blades; Fine Science Tools). Excess abdominal tissue and eggs that remained in the ovipositor were removed, after which the glands were submerged in 50 μL hexane and 200 ng pentadecane as internal standard, as described for the PDMS fibers above. After 30–60 minutes, the glands were removed and the extracts stored at -20°C until analysis.

### Volatile collections

To determine the relationship between gland content and volatile emission of the female pheromone, headspace collections were conducted by pushing air from a compressed air tap through 100 mL glass bottles containing 2–5 virgin females. The airflow was controlled at 0.4 L/minute in each bottle. The Teflon tubings were connected by using stainless steel adapters (IQSG-M54; Jenpneumatik, Jena, Germany). The incoming air was purified by an activated charcoal filter before entering the bottles. Pheromones were trapped using Porapak-Q packed volatile collection traps obtained from Volatile Collection Trap LLC (FL, USA). The traps consisted of a borosilicate glass tube (1/4” OD X 3”) with a 325 mesh Stainless Steel 316 screen and were packed with 20 mg Porapak-Q (Grace Discovery Sciences). The Porapak-Q was held in place with a borosilicate glass wool plug and a PTFE -Teflon compression seal. Pheromones emitted from 2–5 calling females were collected for 2–3 hours between the 3^rd^ and 7^th^ hour of scotophase for Hs, and between the 4^th^ and 8^th^ hour of scotophase for Hv, as we observed that females called most actively in the bottles during these hours. The Porapak-Q traps were eluted using 200 μL hexane, containing 200 ng pentadecane as internal standard. The last drops remaining in the trap were pushed out with a gentle flow of N_2_. Between volatile collections, the traps were cleaned with 2 mL dichloromethane (Rotisolv HPLC; Carl Roth) and 1 mL hexane. Traps were dried using a gentle flow of N_2_. Before the start of each sampling, a 200 μL hexane eluate was taken from every Porapak-Q trap and analysed to confirm that the traps were clean. We refer to the pheromone samples collected by this method as “volatile collections”. The 200 μL eluates were subsequently processed the same way as the PDMS rubs and gland extracts. Directly after sampling, the glands of all females used in the volatile experiments were excised and extracted as described above. Hs glands were thus extracted between the 5^th^ and the 7^th^ hour of scotophase, and Hv glands between the 6^th^ and the 8^th^ hour of scotophase. After each volatile collection, the bottles were cleaned with detergent (Labosol S; NeoLab, Heidelberg, Germany) and rinsed thoroughly with demineralized water, dried in a 60°C oven, after which clean air was pushed through the setup for at least 1 hour before using the bottles for new volatile collections. Blank samples from bottles without moths were taken to confirm that the bottles were clean.

### GC analysis

For pheromone analysis, the volume of all extracts was reduced to 1–2 μL under a gentle stream of nitrogen. To prevent evaporation, the 1–2 μL samples were taken up together with 1–2 μL octane (Anhydrous 99+%; Sigma-Aldrich, Saint Louis, MO, USA) with a 10 μL syringe (701SN 26S GA 2” needle; Hamilton, Bonaduz, Switzerland). The total volume of 2–4 μL was placed in a 50 μL glass insert (27x4 mm; Chromatographie Zubehör Trott, Kriftel, Germany), which was placed in a metal spring (35x5mm; Grace Discovery Sciences) within a 2 mL glass crimp vial (Screening Devices BV, Amersfoort, The Netherlands), capped with an 11 mm aluminum crimp cap and a PTFE (tetrafluoroethylene) septum (Screening Devices BV). The extracts where then injected with an Agilent 7693A Automatic Liquid Sampler into a splitless inlet of a 7890A gas chromatograph (GC; Agilent Technologies, Santa Clara, CA, USA). Between samples, the syringe was cleaned by flushing 10x with acetone (Rotisolv HPLC; Carl Roth) and 10x with hexane.

The GC was equipped with an Agilent DB-WAXetr (extended temperature range) column of 30 m x 0.25 mm x 0.25 μm coupled with a flame ionization detector (FID) at 250°C. The program was as follows: 60°C (hold for 2 minutes) to 180°C (30°C/minute), followed by an increase of temperature to 230°C (5°C/minute). Between samples, the column was heated to 245°C for 15 minutes (20°C/minute). Before and after every series of injections, we injected a blend of authentic standards (Pherobank) of all Hv and Hs pheromone compounds to check the retention times and identify the compounds in the extracts. Areas under the pheromone peaks were determined using Agilent ChemStation (version B.04.03).

### Data analysis

The recovery yield of synthetic pheromone compounds from aluminum foil after 1, 2, and 4 minutes was expressed as a percentage of the total amount present in the control synthetic pheromone blend. The net amount of each compound was calculated relative to 200 ng pentadecane internal standard. To determine the repeatability of the pheromone composition at a rubbing time, the ratio of each compound relative to Z11-16:Ald was calculated. Differences between rubbing times were analyzed using a one-way ANOVA, with separation of means using Tukey’s adjustment for multiple comparisons.

As the total amount of pheromone varies greatly among female moths in general, even within treatments [5,52], and the ratio of the pheromone components is crucial for the attraction of males [1], the effect of the sampling methods on the percentage of each compound in the female pheromone blend was tested by setting the total amount of pheromone at 100%, after which the values were log10 transformed to stabilize the variance. The net amount of pheromone was calculated relative to the 200 ng pentadecane internal standard. A multivariate ANOVA (MANOVA) was used to determine overall differences in pheromone composition between the three different sampling techniques. All pheromone compounds were then compared separately by a univariate ANOVA with separation of means using Tukey’s adjustment for multiple comparisons. All statistical analyses were conducted in IBM SPSS Statistics version 21.

For every pheromone sample, we calculated the ratio of each pheromone compound relative to Z11-16:Ald. We subsequently determined the correlations between the ratios of the PDMS rubs and the corresponding gland extracts. To determine the correlation between the ratios of the volatile collections and the corresponding gland extracts, we used the average of the gland extracts of 2–5 females in each bottle. Spearman's rank correlation coefficients were calculated and plotted in SigmaPlot version 13 (Systat Software Inc., San Jose, CA, USA).

## Results & Discussion

The usefulness of direct-contact SPME in moth chemical ecology has already been demonstrated in a number of studies [16,20]. However, we introduce the use of cheap, disposable, PDMS-coated fused silica optical fibers to overcome the disadvantages of SPME fibers. By using disposable PDMS fibers, analytes can be extracted in hexane in the same way as gland extracts, which eliminates the need for immediate sample analysis, and allows for sampling large numbers of individuals simultaneously. This could be particularly useful for collecting large numbers of samples in the field, as the fibers can be wrapped in aluminum foil (e.g. [26]) and brought to the lab where they can be extracted in hexane. In addition, the hexane extracts of the disposable PDMS fibers can be used for multiple analyses. Also, the disposable PDMS fibers can be cut into custom lengths for specific purposes. To further simplify our method, the fibers could probably also be cleaned by rinsing for 10 minutes in analytical grade methanol and twice in Millipore grade water, following Mayer et al [38], although we did not try this.

**Fig 1:**
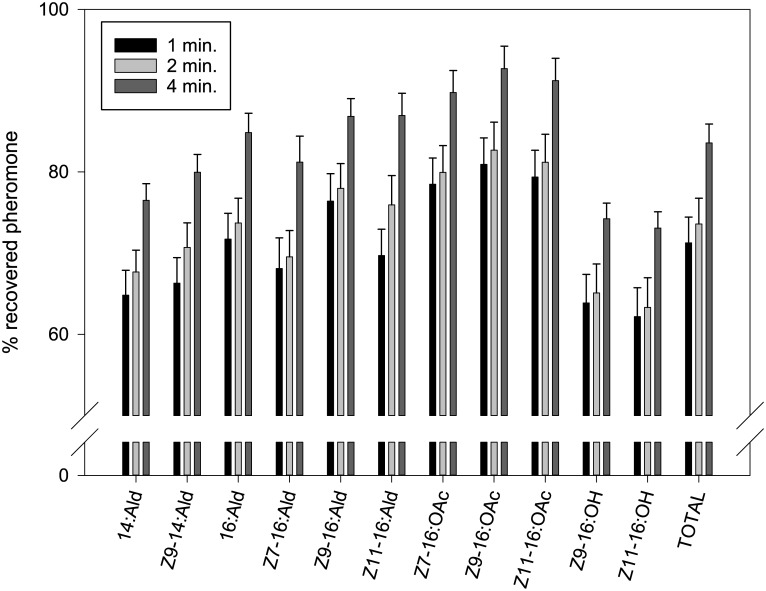
Percentage (mean ± SEM) of synthetic pheromone recovered from aluminum foil after 1 minute (n = 10), 2 minutes (n = 9), and 4 minutes (n = 10) of rubbing with a disposable PDMS fiber. Percentages were calculated relative to the average amount of each compound (set at 100%) present in 2 μL of a synthetic pheromone blend (n = 9).

By rubbing with disposable PDMS fibers, we collected all pheromone compounds of Hs and Hv from aluminum foil (Fig 1). The recovery yields of synthetic compounds from aluminum foil ranged from 62–81% after 1 minute of rubbing, to 73–93% after 4 minutes (Fig 1). The net amount of each pheromone compound is given in S1 Table. We found significant differences between the ratios in samples collected after 1 and 2 minutes of rubbing for Z9-16:Ald, Z7-16:OAc, Z9-16:OAc, and Z11-16:OAc (*P* = 0.007, 0.008, 0.014, and 0.016, respectively), but not between 2 and 4 minutes of rubbing. Therefore, we rubbed the moth glands for at least 2 minutes. However, compared to the control blend (synthetic pheromone blend directly added to hexane), the ratios of C14 aldehydes and the alcohols differed significantly from the 4 minute rubs (14:Ald *P* = 0.002, Z9-14:Ald *P* = 0.004, Z9-16:OH *P* = 0.006, and Z11-16:OH *P* = 0.004), indicating that these compounds were taken up the least effective by the disposable PDMS fiber (Fig 1).

**Fig 2:**
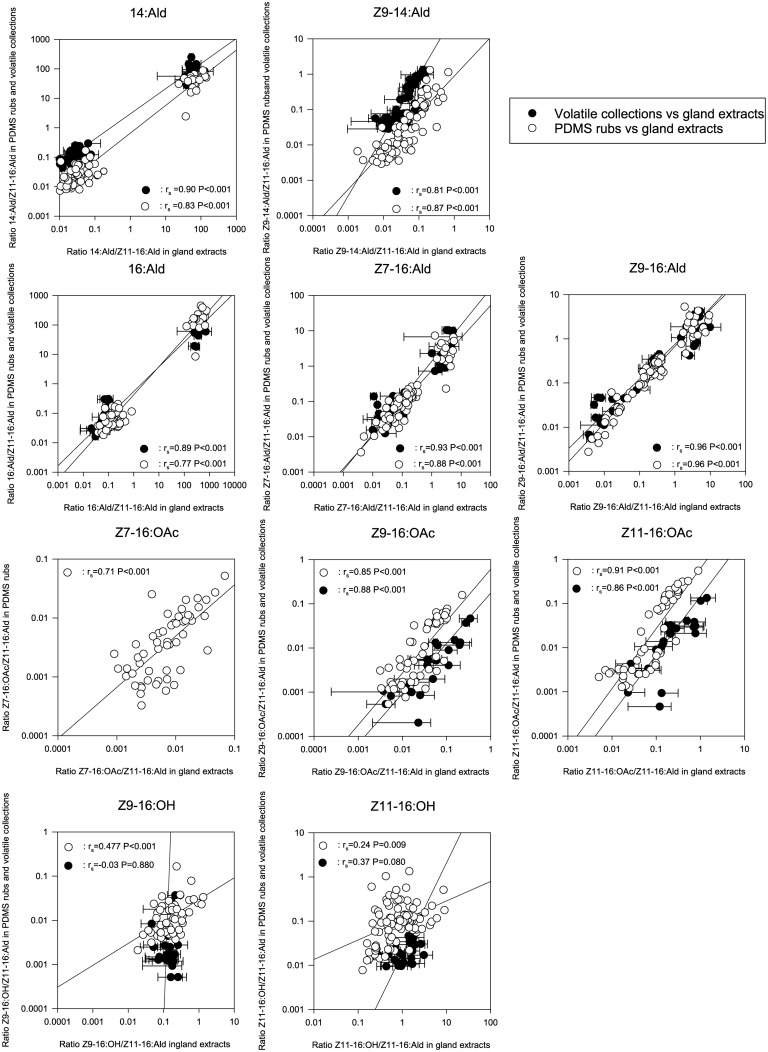
Correlations of ratios of pheromone compounds (relative to the major sex pheromone component Z11-16:Ald) between PDMS rubs and gland extracts (○) and between volatile collections and gland extracts (●). Spearman’s rank correlation coefficients (r_s_) and corresponding significances (*P*) are given for PDMS rubs and volatile collections separately. Gland extracts corresponding to the volatiles represent the mean ratio (± SEM) of 2–5 females from which the volatiles were collected simultaneously. n = 95 for PDMS rubs and n = 50 for volatile collections.

**Fig 3:**
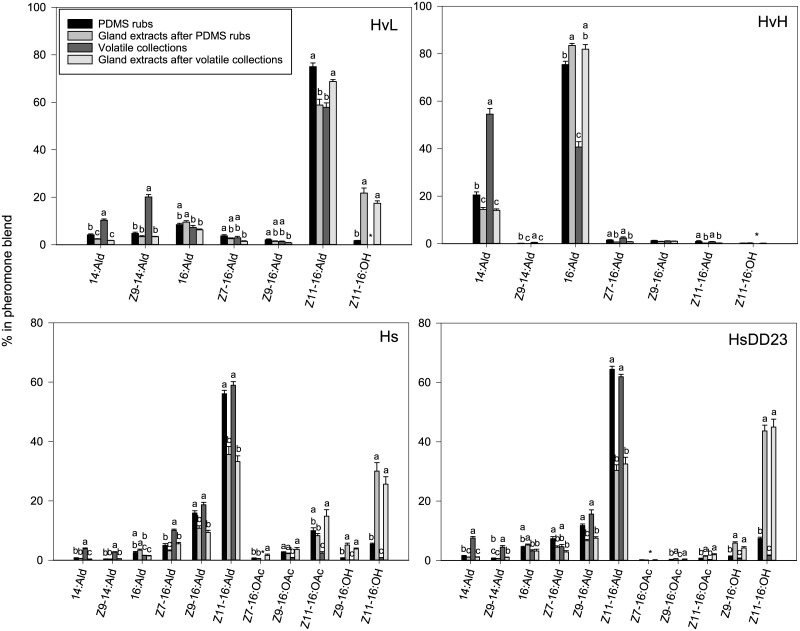
Percentage (mean ± SEM) of each pheromone compound of the total amount of pheromone in PDMS rubs, volatiles collections and the corresponding gland extracts. For PDMS rubs, HvH: n = 20, HvL: n = 21, Hs: n = 21, and HsDD23: n = 32. For volatile collections, HvH: n = 14, HvL: n = 13, Hs: n = 13, and HsDD23: n = 11. Different letters indicate significant differences between sampling techniques (*P* < 0.05). An asterisk (*) indicates that the percentage of the compound could not be reliably determined.

For most compounds in the pheromone blend of female moths, there was a high positive correlation of the ratios relative to Z11-16:Ald between the PDMS rubs and the corresponding gland extracts, as well as between the volatile collections and the corresponding gland extracts (Fig 2). Spearman’s rank correlation coefficients for the aldehydes and acetate esters ranged from 0.71 to 0.96 for PDMS rubs, and from 0.81 to 0.96 for volatile collections. Furthermore, we found that the percentages of the compounds in the blends were comparable between the three techniques (Fig 3). Representative chromatograms of the pheromone blend of Hs for each sampling technique are given in S4 Fig. However, a significant difference was found in the overall pheromone composition of all lines between sampling techniques (*P* < 0.001).

Although the calculated net amount of pheromone is highly variable between individuals, we found consistent differences between the sampling techniques and between Hv and Hs (S2 Table and S5 Fig). In HvH, significantly less pheromone was sampled by PDMS rubs compared to the corresponding gland extracts, whereas in Hs there was no difference between PDMS rubs and gland extracts. Probably, the secretion rate of Hs is higher compared to Hv, as was earlier shown by Heath et al. [5]. The total amount of volatiles collected was significantly lower compared to the corresponding gland extracts and the PDMS rubs.

Some of the differences that we found between the three techniques may have resulted from the experimental procedure. Volatiles were compared with the average pheromone composition of a pool of 2–5 moths, which could be a potential source of error, as it is unlikely that all moths contributed equally to the volatile collections. Sampling time could be an additional source of error, as volatiles were always collected during the 2–3 hours prior to gland extracts. Some of the differences between gland extracts after PDMS rubs and after volatile collections could be explained by the fact that the secreted pheromone on the outside of the gland was mostly removed by PDMS rubbing, whereas the secreted pheromone was still present after volatile collections. Differences that we found between the three methods may also be explained by the chemical properties of the different compounds. Below we describe these differences per class of pheromone compound, viz. aldehydes, acetate esters, and alcohols.

### Aldehydes

In both species, the highest percentages of C14 aldehydes were detected in volatile collections (Fig 3), which were lower in PDMS rubs and lowest in the gland extracts, at least in Hv (Fig 3). The C16 aldehydes showed fewer consistent differences between the sampling techniques. Unsaturated C16 aldehydes were generally detected in higher percentages in volatile collections and PDMS rubs than in the corresponding gland extracts in all four lines, with the exception of HvL (Fig 3). Saturated 16:Ald is the most abundant pheromone compound in HvH, and was found in a lower percentage in volatile collections and PDMS rubs compared to the gland extracts in these females, whereas it was higher in PDMS rubs than in volatile collections in both HvL and HvH (Fig 3).

As we calculated the percentages by setting the total amount of pheromone at 100%, the percentage of C16 aldehydes depended on the differences in the percentages of the other compounds, particularly the C14 aldehydes and alcohols. For example, the percentages of C16 aldehydes in the Hs and HsDD23 pheromone blends were higher in PDMS rubs and volatile collections compared to the gland extracts, which is likely a consequence of the lower percentage of alcohols in PDMS rubs and volatile collections (Fig 3). In Hv, this effect was partly compensated by a higher percentage of C14 aldehydes in volatiles and PDMS rubs.

C14 aldehydes were relatively higher in the volatile collections and PDMS rubs than in gland extracts. This could be explained by the fact that volatility decreases with increased carbon chain length [53]. This was especially apparent in the volatiles of HvH, where a higher percentage of 14:Ald coincided with a lower percentage of 16:Ald. A shorter chain length could potentially also have an effect on the cuticular permeability and secretion rate of pheromone compounds. This may explain why the percentage of 14:Ald was also higher in PDMS rubs than in gland extracts (Fig 3), despite lower retrieval yields of C14 aldehydes by the PDMS fibers (Fig 1). Interestingly, the C14 aldehydes were not detected in volatile collections in Hs in previous studies [5].

### Acetate esters

The retrieval yields of acetates by PDMS rubbing on aluminum foil were high, showing that the PDMS fibers collected the acetates well (Fig 1). Due to the minute amounts, the percentage of Z7-16:OAc could not be reliably determined in the volatile collections, and was therefore excluded from the analyses. The other two acetates (Z9-16:OAc and Z11-16:OAc) in the gland extracts of Hs (4–42% of the total pheromone blend) and HsDD23 (<3% of the total pheromone blend) were detected in PDMS rubs and volatile collections. The percentage of these acetates was similar in the PDMS rubs and the corresponding gland extracts, although in HsDD23 the percentage of Z11-16:OAc was lower in PDMS rubs than in gland extracts. In fact, in HsDD23, the acetates could barely be detected in PDMS rubs, i.e. on the outside of the gland. In both Hs and HsDD23, the percentage of the acetates was much lower in the volatile collections than in the gland extracts (Fig 3). Possibly, acetates are converted to the corresponding aldehydes or alcohols on the gland surface, as suggested by Teal and Tumlinson [54].

### Alcohols

The Z9-16:OH/Z11-16:Ald and Z11-16:OH/Z11-16:Ald ratios had a negligible or low positive correlation between PDMS rubs and the corresponding gland extracts and no significant correlation between the volatiles and corresponding gland extracts (Fig 2). This was mostly due to the fact that Z11-16:OH could not be reliably detected in PDMS rubs and volatile collections of neither HvH nor HvL. Z11-16:OH was detected in PDMS rubs and volatile collections of Hs and HsDD23, albeit in much lower percentages compared to the gland extracts (Fig 3). In Hs and HsDD23, the percentage of Z11-16:OH was higher in PDMS rubs than in volatile collections, (Fig 3).

The fact that we found Z11-16:OH to be present in large amounts in gland extracts of Hv, whereas it was not reliably detected in the volatiles samples, is consistent with the results of Pope et al. [4] and Heath et al. [5]. The alcohols in gland extracts are likely precursors to the aldehydes and acetates [55], which explains the fact that the percentages of Z11-16:OH and Z11-16:Ald are reversed between the gland extracts and the PDMS rubs and volatile collections. In Hv, the alcohols are possibly converted immediately into aldehydes by alcohol oxidases on or within the cuticle of the pheromone gland [56,57]. In Hs, we did find Z11-16:OH in PDMS rubs and volatile collections, albeit in much lower amounts than in gland extracts, which is again consistent with the findings by Heath et al. [5]. The minor compound Z9-16:OH was detected in gland extracts in small amounts in Hs, similar to the findings by Klun et al. [44], whereas it could not be reliably detected in PDMS rubs and volatile collections, probably due to the low amount. As the corresponding aldehyde, Z9-16:Ald, was released as the second largest peak in PDMS rubs and volatile collections, which suggests a precursor role of the alcohol also in Hs.

Another possible explanation of the low percentage of alcohols in volatile collections is the fact that alcohols are less volatile compared to the corresponding aldehydes and acetates due to strong hydrogen bonding. Hydrogen bonding with silanol (Si-OH) groups on the glass surface could also result in adsorption to the inner walls of the glass bottles that contained the moths [58]. In addition, the polar OH-groups of alcohols likely have a lower affinity to the non-polar PDMS fiber [20].

In conclusion, all compounds known to be present in the pheromone blend of two noctuid moths, *H*. *virescens* and *H*. *subflexa*, were detectable by direct-contact sampling with disposable PDMS fibers, showing high recovery yields and strong repeatability after only 2 minutes of rubbing. The pheromone composition in volatile collections, PDMS rubs, and gland extracts was very similar, although the PDMS rubs resembled the composition of the volatile collections more closely than the gland extracts. Thus, we have developed an easy, cheap, reliable, and non-destructive method to determine the pheromone composition of female moths by rubbing disposable, PDMS-coated fused silica optical fibers over the gland surface and subsequently extracting these fibers in hexane. This method can replace the established destructive method of gland extractions and the labor-intensive volatile collection method, in which it is difficult to sample individual females. To our knowledge, this is the first study comparing direct-contact sampling with gland extractions and volatile collections in insects. The direct-contact method with PDMS coated fused silica fibers is likely also very useful in other systems in which volatiles are emitted from a defined glandular structure.

## Supporting Information

S1 Data (XLSX)

S1 Fig Adapted HP5890A gas chromatograph with specifically designed glass conditioning unit inserted into N_2_ flow. (DOCX)

S2 Fig Air-tight glass container for storing PDMS fibers. (DOCX)

S3 Fig Handing of moth and disposable PDMS fiber during pheromone sampling. (DOCX)

S4 Fig Representative chromatograms of the Hs pheromone composition in A) gland extracts after PDMS rubs B) PDMS rubs C) gland extracts after volatile collections and D) volatile collections. (DOCX)

S5 Fig Amount of pheromone (ng ± SEM) collected from live moths by each sampling method. (DOCX)

S1 Table Amount of pheromone (ng ± SEM) collected from aluminum foil by rubbing with disposable PDMS fibers for 1, 2 or 4 minutes. (DOCX)

S2 Table Amount of pheromone (ng ± SEM) collected from live moths by each sampling method. (DOCX)
