# Supplementary material for: Disposable Polydimethylsiloxane (PDMS)-Coated Fused Silica Optical Fibers for Sampling Pheromones of Moths
Source: PLoS One. 2016 Aug 17;11(8):e0161138. doi: 10.1371/journal.pone.0161138 (PMC4988701; doi:10.1371/journal.pone.0161138)
Supplement: S1 Fig — (DOCX) [file pone.0161138.s002.docx]

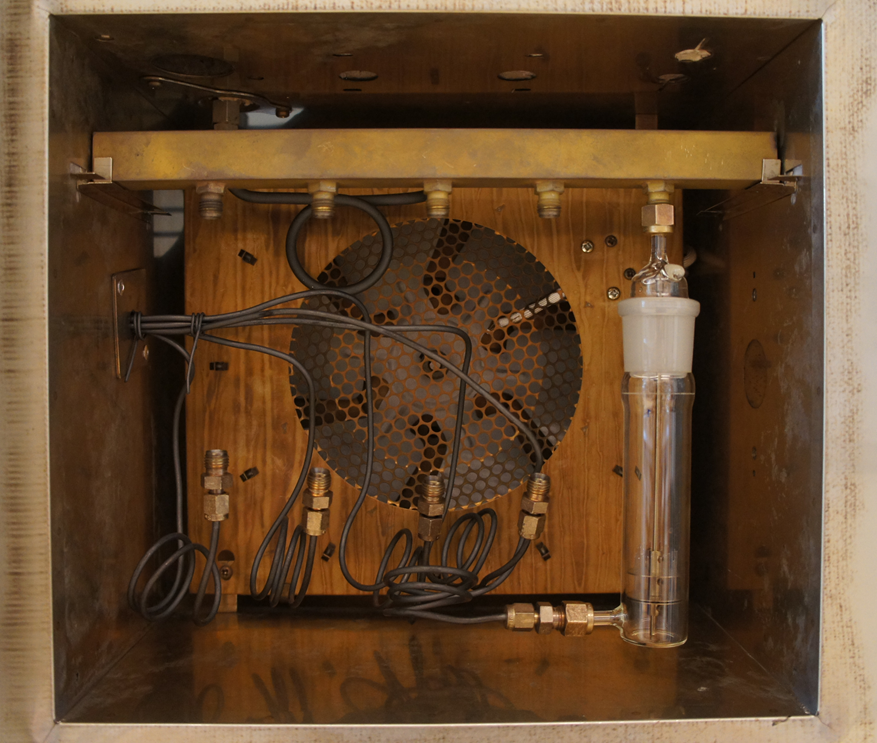


**Fig S1. Adapted HP5890A gas chromatograph with specifically designed glass conditioning unit inserted into N_2_ flow.**
