# Supplementary figures and images for: Disposable Polydimethylsiloxane (PDMS)-Coated Fused Silica Optical Fibers for Sampling Pheromones of Moths
Source: PLoS One. 2016 Aug 17;11(8):e0161138. doi: 10.1371/journal.pone.0161138 (PMC4988701; doi:10.1371/journal.pone.0161138)

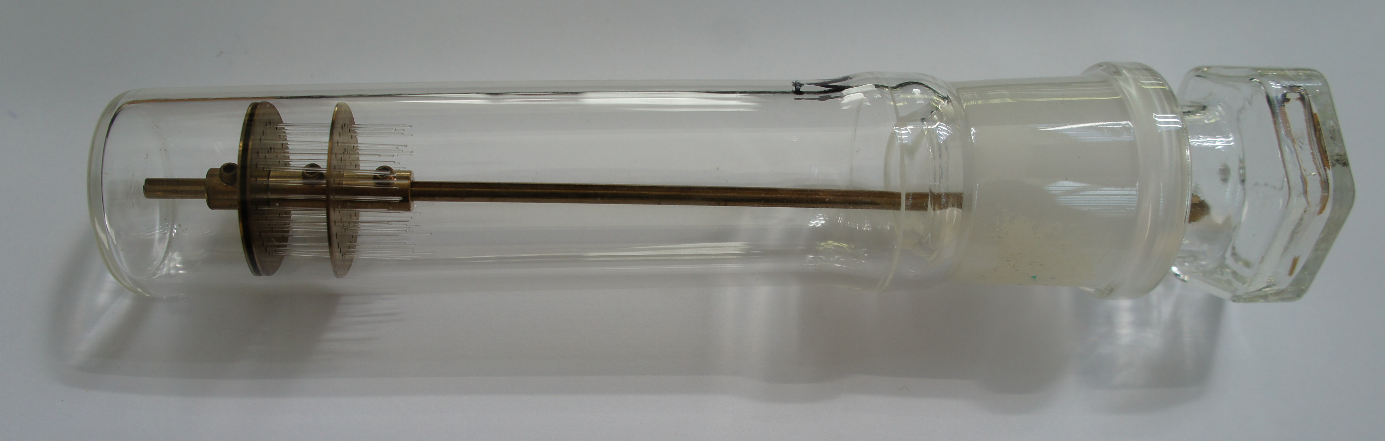


**Fig S2. Air-tight glass container for storing disposable PDMS fibers**

Supplement: S2 Fig — (DOCX) [file pone.0161138.s003.docx]

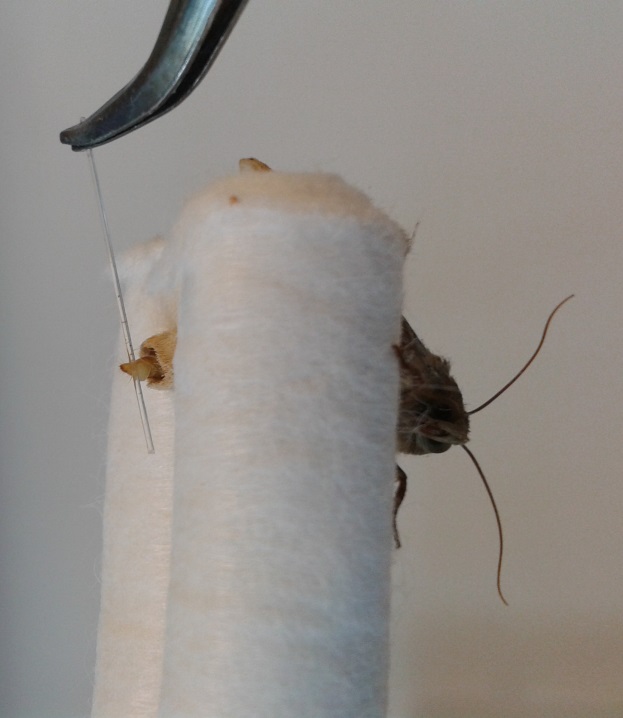


**Fig S3. Handing of moth and disposable PDMS fiber during pheromone sampling.**

Supplement: S3 Fig — (DOCX) [file pone.0161138.s004.docx]
