# Supplementary material for: Disposable Polydimethylsiloxane (PDMS)-Coated Fused Silica Optical Fibers for Sampling Pheromones of Moths
Source: PLoS One. 2016 Aug 17;11(8):e0161138. doi: 10.1371/journal.pone.0161138 (PMC4988701; doi:10.1371/journal.pone.0161138)
Supplement: S4 Fig — (DOCX) [file pone.0161138.s005.docx]

**Fig S4. Representative chromatograms of the Hs pheromone composition in A) gland extracts after PDMS rubs B) PDMS rubs C) gland extracts after volatile collections and D) volatile collections.**
