# Supplementary material for: Disposable Polydimethylsiloxane (PDMS)-Coated Fused Silica Optical Fibers for Sampling Pheromones of Moths
Source: PLoS One. 2016 Aug 17;11(8):e0161138. doi: 10.1371/journal.pone.0161138 (PMC4988701; doi:10.1371/journal.pone.0161138)
Supplement: S5 Fig — (DOCX) [file pone.0161138.s006.docx]

**Fig S5.** Amount of pheromone (ng ± SEM) collected from live moths by each sampling method. Different letters within each treatment indicate significant differences (P < 0.05) between sampling methods, using a univariate ANOVA on log10 transformed values with separation of means using Tukey’s adjustment for multiple comparisons. The amounts for volatile collections and gland extracts after volatile collections represent the average amount of pheromone per female in each bottle.
