# Supplementary material for: Disposable Polydimethylsiloxane (PDMS)-Coated Fused Silica Optical Fibers for Sampling Pheromones of Moths
Source: PLoS One. 2016 Aug 17;11(8):e0161138. doi: 10.1371/journal.pone.0161138 (PMC4988701; doi:10.1371/journal.pone.0161138)
Supplement: S1 Table — (DOCX) [file pone.0161138.s007.docx]

**Table S1.** Amount of pheromone (ng ± SEM) collected from aluminum foil by rubbing with disposable PDMS fibers for 1, 2 or 4 minutes.

| **Compound** | **1 min** | **2 min** | **4 min** | **Control** |
| --- | --- | --- | --- | --- |
| 14:Ald | 20 ± 1 | 21 ± 1 | 24 ± 1 | 31 ± 1 |
| Z9-14:Ald | 15 ± 1 | 16 ± 1 | 18 ± 0 | 23 ± 0 |
| 16:Ald | 26 ± 1 | 27 ± 1 | 31 ± 1 | 36 ± 1 |
| Z7-16:Ald | 17 ± 1 | 17 ± 1 | 20 ± 1 | 25 ± 1 |
| Z9-16:Ald | 22 ± 1 | 23 ± 1 | 25 ± 1 | 29 ± 1 |
| Z11-16:Ald | 22 ± 1 | 24 ± 1 | 27 ± 1 | 32 ± 0 |
| Z7-16:OAc | 25 ± 1 | 25 ± 1 | 29 ± 1 | 32 ± 0 |
| Z9-16:OAc | 25 ± 1 | 26 ± 1 | 29 ± 1 | 31 ± 1 |
| Z11-16:OAc | 25 ± 1 | 26 ± 1 | 29 ± 1 | 32 ± 1 |
| Z9-16:OH | 16 ± 1 | 16 ± 1 | 19 ± 0 | 25 ± 1 |
| Z11-16:OH | 22 ± 1 | 23 ± 1 | 26 ± 1 | 36 ± 1 |
| Total* | 236 ± 9 | 244 ± 9 | 277 ± 7 | 331 ± 5 |

* The total amount increased significantly between 1-4 minutes (*P* = 0.014) but not between 1-2 minutes (*P* = 0.841) and between 2-4 minutes of rubbing (*P* = 0.058), using a univariate ANOVA with separation of means using Tukey’s adjustment for multiple comparisons.
