# Supplementary material for: Disposable Polydimethylsiloxane (PDMS)-Coated Fused Silica Optical Fibers for Sampling Pheromones of Moths
Source: PLoS One. 2016 Aug 17;11(8):e0161138. doi: 10.1371/journal.pone.0161138 (PMC4988701; doi:10.1371/journal.pone.0161138)
Supplement: S2 Table — (DOCX) [file pone.0161138.s008.docx]

**Table S2.** Amount of pheromone (ng ± SEM) collected from live moths by each sampling method.

| **Sampling method** | **HvL** | **HvH** | **Hs** | **HsDD23** |
| --- | --- | --- | --- | --- |
| PDMS rubs | 112 ± 31 | 83 ± 17 | 212 ± 27 | 69 ± 9 |
| Gland extracts after PDMS rubs | 306 ± 112 | 260 ± 57 | 197 ± 26 | 80 ± 6 |
| Volatile collections* | 13 ± 2 | 14 ± 2 | 34 ± 7 | 42 ± 7 |
| Gland extracts after volatile collections* | 199 ± 23 | 260 ± 26 | 245 ± 28 | 145 ± 25 |

*Average amount of pheromone per female in each bottle.
